# Opting out of the Quality and Outcomes Framework (QOF) and impact on practices’ performance

**DOI:** 10.1016/j.puhip.2024.100526

**Published:** 2024-06-24

**Authors:** V. Tzortziou Brown, J. Hayre, J. Ford

**Affiliations:** Wolfson Institute of Population Health, Queen Mary University of London, UK

**Keywords:** QOF, General practice, Incentives, Quality, Outcomes

## Abstract

**Background:**

Financial incentives are being increasingly adopted to help improve standards of care within general practice. However their effects on care quality are unclear. This study aimed to evaluate the impact of practices opting out of the Quality and Outcomes Framework (QOF), a financial incentive scheme in UK general practice.

**Study design:**

A retrospective before and after study of all practices in Tower Hamlets, east London.

**Methods:**

Practices were given an option by local commissioners of opting out of QOF without a financial penalty and instead opting for a locally designed financial incentive scheme that promoted more holistic care. We compared those practices which opted out of QOF to those which continued. We used national, publicly available QOF achievement data from 2016/17 and 2017/18. We undertook a sub-analysis of 16 QOF indicators to better understand the impact of the intervention.

**Results:**

Of the 36 practices in Tower Hamlets, 7 decided to continue with QOF and 29 opted out. The intervention resulted in a small but statistically significant reduction in the total QOF achievement scores of practices which opted out of QOF. The sub-analysis of 16 QOF indicators showed statistically significant reductions in most of achievement scores net of exceptions for the practices that opted out. The differences in performance between the two cohorts of practices became smaller when exceptions were included.

**Conclusions:**

The removal of QOF financial incentives can result in a reduction in achievement of QOF-related indicators but the size of the effect seems to depend on the QOF exception rates. An alternative incentive scheme that promotes a more holistic approach to care seems to be welcomed by general practices.

## What this study adds

• This study assessed the effect of general practices opting out of one of the largest pay-for-performance schemes and revealed reductions in QOF achievement across a range of indicators.
• The study showed that the reductions in QOF achievements became smaller when exceptions were taken into consideration.
• An alternative incentive scheme that promotes a more holistic approach to care seems to be welcomed by practices and may need to be further explored.

## 1 Introduction

Financial incentives, such as pay-for-performance schemes, combined with quality indicators are used in general practice globally to improve care. The United Kingdom's Quality and Outcomes Framework (QOF) represents one of the largest pay-for-performance schemes for general practice in the world. The scheme financially remunerates general practices for delivering interventions and achieving patient outcomes using evidence-based indicators developed by the National Institute for Health and Care Excellence (NICE) [1]. Over 90 % of general practices in England enrol in QOF and it accounts for about 10 % of a practice's income [2].

QOF has resulted in improvements in quality of care, but has also been criticised as promoting a departure from patient-centred, holistic care to a measurable, reductionist approach [3]. In addition, QOF achievement may not give a true reflection of population health, utilising indirect surrogate measures [4] and indicators that meet managerial or policy agendas rather than clinical priorities [5]. The emphasis on single conditions may have disadvantaged the management of multimorbidity which is more common in deprived areas [6], while any financial gains may be reduced when the additional administrative staff required for reaching QOF targets are taken into account [3].

Given some of the criticisms of QOF, practices in Tower Hamlets, east London, were given an opportunity to opt out of QOF and focus on an alternative Network Improvement Scheme (NIS) which included a reduced number of clinical quality indicators and promoted a more person-centred approach to care. The aim of this study was to evaluate the impact of practices opting out of QOF compared to those who continued.

## 2 Methods

We designed a retrospective before and after study of all practices in Tower Hamlets, east London. Practices were given an option by local commissioners of opting out of QOF without a financial penalty and instead opting for a locally designed financial incentives scheme (NIS).

The NIS included a significantly reduced number of QOF clinical targets and the addition of targets related to holistic care. Under the NIS only 33 out of the 77 QOF indicators continued to be incentivised. Of the 44 that were not incentivised, 23 were monitored through dashboard reporting and 21 were made redundant. The full list of targets which continued to be incentivised, were monitored and removed is included in the Supplementary Material.

We compared those practices which opted out of QOF to those which continued. We used national, publicly available QOF achievement data from 2016/17 and 2017/18. NHS Digital uses a General Practice Extraction Service to extract these data directly from practice records and they are published annually. Achievement to target was extracted for each of the practices for 2016/17 and 2017/18 data with and without exceptions.

We undertook a sub-analysis of 16 QOF indicators on common long-term conditions and smoking to better understand the impact of the intervention on QOF performance at practice level.

Data was descriptively analysed to compare the change in performance for those practices which opted out of QOF to those that continued. The statistical significance of the changes in practices’ performance was calculated using the *t*-test (significance was set at 0.05).

## 3 Results

Of the 36 practices in Tower Hamlets, 7 decided to continue with QOF and 29 opted out. The two cohorts of practices were comparable in terms of QOF performance prior to the intervention and had no statistically different results (p = 0.23).

The intervention resulted in a small but statistically significant reduction in the total QOF achievement scores of practices which opted out of QOF (average achievement net of exceptions 88.52 %, standard deviation 7.22, p < 0.0001) while the total QOF achievement of practices who continued with QOF showed a slight non-statistically significant increase (average achievement net of exceptions 94.17 %, standard deviation 4.73, p = 0.45).

### 3.1 Sub-analysis

The sub-analysis of 16 QOF indicators showed statistically significant reductions in QOF achievement scores net of exceptions for the practices that opted out of QOF across all five indicators which continued to be incentivised, five of six indicators which were monitored and four of five indicators which were stopped (Table 1). Statistically significant reductions in QOF achievement scores net of exceptions were also noted across two of 16 indicators for practices which continued with QOF.

**Table 1:** Average achievement across QOF amongst practices which opted out of QOF and those which continued with QOF. Numbers in bold show statistically significant results (T test, p < 0.05). Where * means that patient numbers in some practices were very low (<5) which can influence the validity of the results.

Indicators	Practices opting out of QOF			Practices continuing QOF		
	Average achievement net of exceptions before, after and relative change	Average achievement including exceptions before after and relative change	Average exception rate (%) before, after and relative change	Average achievement before, after and relative change	Average achievement including exceptions before after and relative change	Average exception rate (%) before, after and relative change
**Continued to be incentivised via the new NIS**						
**CHD002** The percentage of patients with coronary heart disease in whom the last blood pressure reading (measured in the preceding 12 months) is 150/90 mmHg or less	**95 %**	**94 %**	1 %	**95 %**	**94 %**	1 %
	**91 %**	**89 %**	2 %	**92 %**	**91 %**	1 %
	**−4%**	**−5%**	57 %	**−3%**	**−3%**	43 %
**DM007** Diabetic patients with last IFCC HbA1c is 59 mmol/mol or less in the prior 12 months.	**67 %**	**61 %**	**8 %**	62 %	**58 %**	5 %
	**61 %**	**58 %**	**5 %**	60 %	**56 %**	7 %
	**−10 %**	**−6%**	**−40 %**	−2%	**−4%**	41 %
**COPD007** COPD patients who have had influenza immunisation in the preceding 1 August to 31 March.	**98 %**	82 %	17 %	96 %	80 %	16 %
	**95 %**	81 %	15 %	96 %	77 %	20 %
	**−3%**	−1%	−8%	0 %	−4%	22 %
**SMOK002** Patients with: CHD, PAD, stroke or TIA, hypertension, diabetes, COPD, CKD, asthma, schizophrenia, bipolar affective disorder or other psychoses with a recorded smoking status in prior 12-months.	**96 %**	**95 %**	0.58 %	96 %	95 %	0.74 %
	**95 %**	**94 %**	0.54 %	96 %	95 %	0.57 %
	**−1%**	**−1%**	−6%	0 %	0 %	−23 %
**SMOK005** Patients with: CHD, PAD, stroke or TIA, hypertension, diabetes, COPD, CKD, asthma, schizophrenia, bipolar affective disorder or other psychoses recorded as current smokers who have a record of an offer of support and treatment within the prior 12-months.	**97 %**	**96 %**	0.47 %	92 %	91 %	0.93 %
	**92 %**	**91 %**	0.49 %	97 %	96 %	0.87 %
	**−5%**	**−5%**	4 %	0 %	0 %	−7%
**Monitored**						
**STIA007** Non-haemorrhagic stroke of history of TIA patients in the prior 12-months on anti-platelet or anti-coagulation treatment.	**98 %**	**91 %**	**7 %**	99 %	91 %	8 %
	**92 %**	**87 %**	**5 %**	98 %	89 %	9 %
	**−6%**	**−4%**	**−34 %**	−1%	−2%	11 %
**DM003** Adult patients with depressions who have been reviewed not earlier than 10 days after and not later than 56 days after the date of diagnosis.	**85 %**	**81 %**	4 %	**82 %**	**81 %**	2 %
	**79 %**	**77 %**	4 %	**76 %**	**74 %**	5 %
	**−7%**	**−6%**	−9%	**−7%**	**−8%**	112 %
**MH008** Women aged ≥25 and ≤ 65 with schizophrenia, bipolar affective disorder and other psychoses with a cervical screening test being performed in the prior 5 years.	89 %	70 %	21 %	85 %	69	18 %
	87 %	70 %	19 %	91 %	70	23 %
	−2%	0 %	−8%	8 %	2 %	24 %
**MH009** Patients on lithium therapy with a record of serum creatinine and TSH in the prior 12-months.	**96 %**	93 %	3 %	92 %	89 %	5 %
	**88 %**	87 %	2 %	90 %	89 %	1 %
	**−8%**	−6%	−38 %	−1%	0 %	−79 %
**CAN003** Cancer patients, diagnosed within the preceding 15 months, with a patient review 6-months after diagnosis.	**96 %**	**83 %**	**14 %**	95 %	78 %	18 %
	**80 %**	**63 %**	**22 %**	97 %	62 %	35 %
	**−17 %**	**−24 %**	**61 %**	2 %	−20 %	97 %
**CS002** Women aged ≥25 and ≤ 65 with cervical cancer screening in the prior 5-years.	**78 %**	**70 %**	10 %	76 %	67 %	11 %
	**77 %**	**69 %**	10 %	75 %	66 %	11 %
	**−2%**	**−2%**	−1%	−1%	−2%	4 %
**Stopped**						
**AF007** Atrial Fibrillation patient with a CHA2DS2-VASC score of ≥2 on anticoagulation therapy.	89 %	82 %	8 %	**87 %**	82 %	6 %
	89 %	82 %	8 %	**92 %**	83 %	9 %
	0 %	−1%	11 %	**5 %**	2 %	36 %
**DM004** Diabetes patients with last measured cholesterol being 5 mmol/l or less.	**86 %**	80 %	6 %	82 %	78 %	4 %
	**84 %**	79 %	6 %	82 %	77 %	6 %
	**−2%**	−1%	−7%	0 %	−2%	51 %
**COPD003** COPD breathlessness review in the preceding 12-months.	**92 %**	**88 %**	**5 %**	89 %	84 %	5 %
	**86 %**	**83 %**	**3 %**	91 %	84 %	8 %
	**−7%**	**−5%**	**−47 %**	3 %	0 %	57 %
**OST002** Patients aged >50 and < 75, with a fragility fracture and osteoporosis diagnosis on DEXA scan, on at least one bone-sparing agent.	**98 %**	**92 %**	6 %	100 %	100 %	0 %
	**96 %**	**66 %**	12 %	86 %	79 %	7 %
	−22 %*	−29 %*	107 %	−14 %*	−21 %*	
**OST005** Patients aged >75, with a fragility fracture, on at least one bone-sparing agent.	**93 %**	89 %	4 %	93 %	93 %	0 %
	**83 %**	77 %	8 %	100 %	88 %	12 %
	−11 %*	−14 %*	104 %	7 %*	−6%*	

The differences in performance between the two cohorts of practices became smaller when exceptions were included in the scores. The largest difference between the two cohorts was on QOF achievement scores net of exceptions for cancer reviews following a new diagnosis which was also statistically significant (average achievement 80 %, standard deviation 21.69 versus 97 %, standard deviation 5.26, p < 0.005). However, this difference reversed and became statistically non-significant when exceptions were included (average achievement 63 %, standard deviation 22.63, versus 62 %, standard deviation 20.91, p = 0.9).

Exception rates decreased for the majority of assessed (10/16) indicators for practices which opted out of QOF (4 of these reductions were statistically significant). Exceptions increased for 13/16 indicators for practices which opted to continue with QOF, although these did not reach statistically significant levels.

## 4 Discussion

The majority of practices opted out of QOF demonstrating that an alternative incentive scheme that advocates for a more holistic approach to care may be welcomed. QOF performance dropped across several indicators amongst practices which opted out of QOF. This finding is in accordance with the results of the evaluation of the Somerset Practice Quality Scheme [7] and a study by Lester et al. [8] which identified an approximate 3 % fall in achievement per year following the removal of incentives. Interestingly, performance dropped across indicators which continued to be incentivised via the NIS and those monitored for practices opting out of QOF. This could be because of inconsistent practice administrative and monitoring arrangements in view of a new incentive scheme during the study period. From an organisational perspective, there is a consensus view that QOF improves data recording; with QOF implementation resulting in a 19.9 % increase in annual recording rates of 5 health indicators [9].

Practices which opted out of QOF tended to have lower exception rates across the assessed indicators. The differences in QOF achievement across the 16 indicators between practices opting in and out of QOF became smaller when exceptions were included in the scores. The available clinical data did not allow an assessment of whether these exceptions were clinically justified or whether they reflected patient choice. There is some evidence that exception reporting is sometimes exploited at the end of the payment year to prevent the practice being penalised financially [10]; therefore, skewing the monitoring benefit of QOF towards compliant patients.

## Strengths and limitations

A strength of our study is the utilisation of 32 general practices serving a population of high deprivation. A further strength is that opting out of QOF could be explored without financial penalty acting as a confounder, since the practices were remunerated through the NIS initiative. The available data on exception reporting lacked information on whether the exception reporting was clinically justified or due to genuine patient preference: providing this information through coded format would permit a better understanding on whether such exceptions are justified in future studies.

## Implications for policy and practice

When operating in a resource constrained environment, it can be challenging to decide whether shifting clinician efforts to those areas of care which are incentivised and publicly reported should take priority over those activities that promote continuity, shared decision making and relational care. Such tensions need to be considered carefully when designing incentive schemes that aim to improve quality of care.

The reporting of exceptions needs to be better understood to ensure it accurately reflects quality of care. QOF targets should not impair patient choice following a shared decision-making conversation.

Evidence from the evaluation of different pay-for-performance schemes internationally shows that they have only had a limited impact on mortality and patient outcomes. It may be that a less biomedical approach to care with a greater emphasis on delivering personalised care and encompassing a wider range of quality improvement activities is preferable to general practices and more successful at improving care quality.

## Ethical approval

Not required.

## Funding

The study did not receive any external funding. VTB and JF received NIHR fellowship funding during part of the evaluation.

## Declaration of competing interest

The authors declare the following financial interests/personal relationships which may be considered as potential competing interests:

VTB and JH have no conflicts of interest to declare.

JF is an editor of the Journal Public Health in Practice but won't be involved in the editorial review of the article.
